# How do Swiss general practitioners agree with and report adhering to a top-five list of unnecessary tests and treatments? Results of a cross-sectional survey

**DOI:** 10.1080/13814788.2017.1395018

**Published:** 2017-11-23

**Authors:** Kevin Selby, Jacques Cornuz, Christine Cohidon, Jean-Michel Gaspoz, Nicolas Senn

**Affiliations:** ^a^ Department of Ambulatory Care and Community Medicine, University of Lausanne Lausanne Switzerland; ^b^ Division of Research, Kaiser Permanente Northern California Oakland CA USA; ^c^ Institute of Family Medicine, University of Lausanne Lausanne Switzerland; ^d^ Division of Primary Care Medicine, Department of Community Medicine, Primary Care and Emergency Medicine, Geneva University Hospitals and Faculty of Medicine, University of Geneva Geneva Switzerland

**Keywords:** Clinical decision-making, general practice, medical overuse, proton pump inhibitors, rational use of medications

## Abstract

**Background:** In 2014, the ‘Smarter Medicine’ campaign released a top five list of unnecessary tests and treatments in Swiss primary care, such as imaging for acute low-back pain and long-term prescribing of proton pump inhibitors.

**Objectives:** Measure general practitioners’ (GPs) agreement with the recommendations and self-reported adherence.

**Methods:** Cross-sectional, online survey of GPs in the ‘Swiss primary care active monitoring’ (SPAM) network, which assessed awareness of ‘Smarter Medicine’ and views on each recommendation. Questions included whether the clinical situation is common, whether the recommendation is followed, whether GPs agree with the recommendation and reasons why the recommendation would not be followed.

**Results:** One-hundred-and-sixty-seven of 277 GPs from the SPAM network participated (60%), of which 104 (62%) knew of ‘Smarter Medicine’, including 79% in German areas, 49% in French areas and 38% in Italian areas (*P* < 0.001). Agreement with the five recommendations was high, with scores around nine out of 10. The proportion saying they typically follow each recommendation was 68 to 74%, except not continuing long-term PPI prescriptions without attempting dose reduction, with only 34%. Common reasons for not following the recommendations were patient or other provider requests and situations that might suggest the need for more aggressive care.

**Conclusion:** Two years after the launch of the campaign, awareness and acceptance of ‘Smarter Medicine’ appear to be high among Swiss GPs. By self-report, the recommendations are adhered to by most of the respondents but there may be room for improvement, especially for long-term PPI prescriptions.

KEY MESSAGES

- The ‘Smarter Medicine’ campaign adopted a top five list of unnecessary tests and treatments for Swiss primary care.
- General practitioners agree with all of the recommendations but report low adherence to the recommendation to down-titrate or stop chronic prescriptions of proton pump inhibitors.

## Introduction

There has been an international drive by physician societies to identify unnecessary tests and treatments. Under the banner of the ‘Choosing Wisely’ campaign, initiatives have appeared in multiple countries, including the United States, Canada, Netherlands, UK, Australia and Germany [1,2]. The campaigns encourage ‘evidence-based conversations about what tests and treatments may not benefit [patients] and could cause harm [1].’

While the Swiss healthcare system does well on global indicators of quality, it has the second-highest per-capita expenditures in the world. With a fee-for-service payment structure and very high out-of-pocket costs, there is increasing public pressure to lower costs [3]. The Swiss Society of General Internal Medicine (SSGIM), which represents general practitioners (GPs) in Switzerland, launched the ‘Smarter Medicine’ campaign (http://www.smartermedicine.ch) in May 2014 with a list of five unnecessary tests and treatments [4,5]. The five items address several facets of ‘too much medicine’ in ambulatory general internal medicine [6]: imaging for acute, uncomplicated low-back pain; prostate cancer screening without discussing the risks and benefits; antibiotics for uncomplicated upper respiratory tract infections; preoperative chest radiography without clinical suspicion of pathology; and long-term treatment with proton pump inhibitors without titrating to the lowest effective dose.

National experts (35) chose the items specifically for the Swiss context, but the list’s impact on community based practice remains uncertain for several reasons. First, the ‘Smarter Medicine’ campaign has depended on effective diffusion of its message through the traditional channels by which Swiss physicians acquire new information. Extensive research shows that recommendations alone do not reliably change physician behaviour [7]. Second, while physicians acknowledge that waste exists in our healthcare system, they may be doubtful of their personal responsibility and what effect they can have on costs [8]. Third, the data showing that the items in the list are commonly overused, such as antibiotic use in upper respiratory tract infections (URIs) and imaging for low-back pain [9,10], come primarily from Anglo-Saxon countries. The actual pattern of overuse of Switzerland remains virtually unknown, except for data showing regional variations and global indicators such as antibiotic prescriptions per capita [11–13]. Finally, efforts to change practice will likely need to be long-term and multifaceted, and must be guided by an understanding of the underlying factors leading to prescribing ‘too much medicine’ [14]. Possibilities include a lack of updated knowledge, physician routine, patient requests, inappropriate incentives, or that physicians feel that guidelines are not applicable for certain subgroups of patients [15].

The aims of this study were to assess general practitioner (GP) awareness of the ‘Smarter Medicine’ campaign, the relevance of the items to their daily practice, reasons why they might *not* follow the recommendations, and their agreement with the recommendations as published. We assessed these questions among physicians participating in a practice-based research network.

## Methods

### Setting and design

Both GPs and general internists provide primary care in Switzerland, are represented by the SSGIM and are referred to as GPs. Between 2012 and 2014, a SSGIM committee identified low-value healthcare activities for a Swiss top five list, as previously published [5]. After a literature review, an online Delphi process was used with 35 practicing GP-experts to rate existing recommendations and offer new ones. The committee members reported no conflicts of interests.

For the current study, we conducted an online survey with members of the Swiss primary care active monitoring (SPAM) network, a national practice-based research network. Results were collected between August 2015 and May 2016, approximately two years after the launch of the ‘Smarter Medicine’ top five list. The SPAM network was created in 2012 with 200 members to monitor the practice patterns of Swiss GPs [16]. To augment the membership and replace physicians leaving the network, a further 3000 GPs were invited to participate by mailed invitation in 2015, increasing the network to 277 members. These 277 members received a questionnaire that included a section about ‘Smarter Medicine’ as part of the SPAMPREV study. A Swiss Academy of Medical Sciences funded the study about preventive medicine. Approval for this study was obtained from the ethics committee of the Canton of Vaud.

### Survey instrument

The electronic survey instrument contained three sections. First, GPs were asked demographic questions including the postal code of their practice (to determine if the practice was in a rural area) and in-practice availability of a pharmacy, radiology, and laboratory equipment.

Second, GPs were asked if they were aware of the ‘Smarter Medicine’ campaign, ‘Choosing Wisely’ and the National Institute for Health and Clinical Excellence (NICE) ‘do not do’ lists [1,17], based on a question from a American Board of Internal Medicine (ABIM) survey [18]. They were presented with each of the five items from the ‘Smarter Medicine’ list and asked how frequently they encountered the scenario presented in their practice; how often, in that situation, they followed the recommendation; reasons why they might *not* follow the recommendation; and whether they agreed with the recommendation as written in the ‘Smarter Medicine list.’ The physician practices questions were based on the ABIM survey and the methodology used by the National Physicians Association to validate their top five lists [19]. The physician agreement question used the same phrase and 10-point Likert scale as used in for the original ‘Smarter Medicine’ list [5]. The survey was first developed and tested locally in French and is available upon request. It was then translated into German and Italian, and administered using online software from SurveyMonkey^®^.

### Statistical analysis

For each response, we calculated frequencies and means as appropriate. A Poisson regression model was built on the primary outcome of how many of the five recommendations GPs reported not following more often than ‘rarely or never’. Answering ‘rarely or never’ was considered following the recommendation, except for discussing prostate cancer screening, where discussing ‘most of the time’ before screening was considered following the recommendation. Provider characteristics (sex, age, country of medical training, experience in practice) and practice characteristics (size, location and having an in-practice pharmacy, laboratory and/or radiology equipment) were included as independent variables as well as awareness of ‘Smarter Medicine’ and having mean acceptance of greater than eight for the five recommendations. Univariate analyses were first performed and variables significant to a *P*-value less than 0.20 were retained for multivariate modelling. Analyses were performed using STATA version 14 (StataCorp).

## Results

Of the 277 physicians participating in the SPAM network, 167 completed the online survey (response rate 60%). Characteristics of the physicians participating are shown in Table 1. As seen in Table 2, 62% of respondents were aware of the Swiss ‘Smarter Medicine’ campaign. Significantly, more respondents were aware of ‘Smarter Medicine’ in German-speaking areas (79%) than in French and Italian-speaking areas (49 and 38%, respectively).

**Table 1.** Characteristics of respondents from the Swiss primary care active monitoring network (*n* = 167).

Characteristic	
Sex (women)	50 (30%)
Mean age (±SD)	54 (±8.9)
Language area	
German	90 (54%)
French	61 (37%)
Italian	14 (8%)
Country of medical training (Switzerland)	151 (90%)
Years of experience in practice (±SD)	18 (±11)
Practice size	
Solo practice	46 (28%)
2 to 4 physicians	105 (63%)
5 or more physicians	16 (10%)
Practice in rural area	43 (26%)
Average consultation <20 min	70 (42%)
In practice:	
Pharmacy	69 (41%)
Laboratory	143 (86%)
X-ray equipment	106 (63%)

**Table 2.** Awareness of campaigns to decrease overuse among general practitioners (*n* = 167).

Question	*n* (%)
Have you heard about the campaign ‘Smarter Medicine’	
Yes	104 (62%)
No	45 (27%)
I don’t know	11 (7%)
Have you heard about the campaign ‘Choosing Wisely’	
Yes	95 (57%)
No	63 (38%)
I don’t know	2 (1%)
Have you heard about the ‘do not do’ lists?	
Yes	77 (46%)
No	73 (44%)
I don’t know	8 (5%)

The frequency with which GPs encounter the scenarios of the ‘Smarter Medicine’ top five list and the proportion that report rarely going against the recommendations are shown in Table 3. The proportion of respondents saying that they rarely or never go against each recommendation (or answered ‘most of the time’ for the positively phrased question of discussing prostate cancer screening before performing the PSA) ranged from 34% for the recommendation to not continue PPIs, or decrease the dose, to 74% for performing preoperative chest X-rays. Physician agreement with the recommendations on a 10-point Likert scale (with 10 most likely to agree) is also shown in Table 3. The agreement scores were high, around nine out of 10.

**Table 3.** Proportion of physicians who encounter clinical scenarios often or very often^a^, who rarely or never go against each ‘Smarter Medicine’ recommendation, and mean agreement with recommendation^b^.

Clinical scenario	Category	Total (*n* = 167)
1. Patients with non-specific low-back pain	Encountered often or very often^a^	138 (83%)
	Rarely or never get imaging	114 (68%)
	Mean agreement with recommendation not to get imaging for non-specific low-back pain (0–10)^b^	9.0 (±1.9)
2. Prostate cancer screening using prostate-specific antigen test (PSA)	Encountered often or very often^a^	97 (58%)
	Discuss most of the time prior to screening	116 (69%)
	Mean agreement with recommendation to discuss before screening (0–10)^b^	8.9 (±1.7)
3. Upper respiratory tract infections without signs of complications	Encountered often or very often^a^	152 (91%)
	Rarely or never prescribe antibiotics for these infections	113 (68%)
	Mean agreement with recommendation not to prescribe antibiotics for these infections (0–10)^b^	9.1 (±1.5)
4. Patients without lung pathology for preoperative assessment	Encountered often or very often^a^	79 (47%)
	Rarely or never request a chest X-ray	124 (74%)
	Mean agreement with recommendation not to request preoperative chest X-rays (0–10)^b^	9.2 (±1.4)
5. Long-term use of proton pump inhibitors without confirmed pathology	Encountered often or very often^a^	98 (59%)
	Rarely or never continue medication without lowering dose	56 (34%)
	Mean agreement with recommendation to not continue medication without lower dose (0–10)^b^	8.9 (±1.4)

^a^ Often defined as weekly and very often as very often.

^b^ On a 10-point Likert scale, from 0 (complete disagreement) to 10 (complete agreement).

The most frequent reasons for which physicians might not follow each given recommendation are shown in Table 4. The patient’s request was important for imaging in low-back pain (67%) and providing antibiotics for URIs (73%), while the surgeon’s request was important when performing preoperative chest X-rays (68%). The number responding that this question was not applicable to them as they always follow the recommendation ranged from 20% for not continuing proton pump inhibitors, to 56% saying that they always discuss prostate cancer screening prior to testing. The responses that physicians lack time to follow the recommendations are attempting to avoid a medical error, are attempting to gain patient trust, or perform these tests for new or unknown patients were rarely chosen (not shown).

**Table 4.** Reasons why physicians go against the recommendations that received 12 or more positive responses, with several responses possible (*n* = 167).

Recommendation	Reasons for not following recommendation	GPs responding yes (%)
1. Do not obtain imaging studies for patients with non-specific low-back pain	Request or insistence of patient	89 (67%)^a^
	Desired information from imaging	28 (21%)^a^
	To increase bond with the patient	17 (13%)^a^
	Not applicable as I always follow this recommendation	35 (21%)
2. Do not perform prostate cancer screening without a discussion	PSA already ordered previously	45 (62%)^a^
	Patients don’t want to discuss	15 (21%)^a^
	I don’t have the time	12 (16%)^a^
	Not applicable as I always follow this recommendation	94 (56%)
3. Do not prescribe antibiotics for uncomplicated respiratory tract infections	Prescribed for patients at high risk of complications	91 (73%)^a^
	Request or insistence of patient	58 (46%)*
	For infections lasting more than 10 days	54 (43%)*
	Not applicable as I always follow this recommendation	42 (25%)
4. Do not obtain chest radiography in the absence of suspected lung pathology	Requested by surgeon	78 (68%)^a^
	Not applicable as I always follow this recommendation	53 (32%)
5. Do not continue long-term use of proton pump inhibitors without titrating to the lowest dose needed	PPIs continued to avoid recurrent symptoms	61 (46%)^a^
	I allow patients to decide whether to continue medication	36 (27%)^a^
	PPIs started by another physician	30 (22%)^a^
	PPIs continued to avoid complications	18 (13%)^a^
	Not applicable as I always follow this recommendation	33 (20%)

^a^ Those responding ‘not applicable’ excluded from totals for percentages to other responses.

There were 159 GPs with complete responses to all questions regarding whether the recommendations are followed, of whom 2 (1%), 8 (5%), 27 (17%), 51 (32%), 49 (31%) and 22 (14%) reported following zero, one, two, three, four and five recommendations respectively. Multivariate Poisson regression found a significant correlation between both average agreement scores with the recommendations lower than eight out of 10 and presence of an in-practice pharmacy and following fewer recommendations (Table 5).

**Table 5.** Factors associated with following fewer of the ‘Smarter Medicine’ Top five recommendations (*n* = 159)^a^.

Characteristic	Univariate incidence rate ratio (95%CI)^b^	*P*	Multivariate incidence rate ratio (95%CI)^b^	*P*
Sex (women)	0.98 (0.77–1.25)	0.86	–	
Age, years	1.00 (0.99–1.01)	0.91	–	
Language area, German	0.75 (0.62–0.92)	0.01	1.05 (0.84–1.30)	0.69
Country of medical training, outside Switzerland	1.08 (0.73–1.61)	0.69	–	
Experience in practice, years	1.00 (0.99–1.01)	0.90	–	
Practice size, five or more physicians	0.82 (0.56–1.19)	0.30	–	
Practice location, rural	1.01 (0.81–1.26)	0.91	–	
In-practice pharmacy, yes	**0.70 (0.57–0.86)**	**<0.01**	**0.71 (0.57–0.88)**	**0.002**
In-practice laboratory, yes	0.94 (0.66–1.35)	0.74	–	
In-practice radiology, yes	0.93 (0.75–1.16)	0.53	–	
Knows of ‘Smarter Medicine’	0.91 (0.73–1.14)	0.42	–	–
Mean agreement with recommendations ≤8^c^	**1.62 (1.34–1.96)**	**<0.01**	**1.60 (1.33–1.94)**	**<0.001**

^a^ Defined as responding ‘nearly always’ for discussing prostate cancer screening prior to testing and ‘rarely or never’ to the other four clinical scenarios.

^b^ Incidence rate ratio gives the ratio for following one additional recommendation.

^c^ Mean agreement for individual GPs with the five recommendation statements.

## Discussion

### Main findings

In this cross-sectional survey of practicing GPs from throughout Switzerland, most physicians were aware of the ‘Smarter Medicine’ campaign and agreed with its recommendations. The only recommendation with less than two-thirds reporting that they ‘rarely or never’ go against the recommendation was to decrease the dose or stop prescriptions for proton-pump inhibitors. Reasons for not following the recommendations varied, but patient request and desire for a test’s results were more important drivers than lack of time or fear of malpractice.

The wide recognition of the ‘Smarter Medicine’ campaign in our study was higher than similar studies among community physicians [18,20] but lower than among academic emergency medicine doctors [21]. It is likely that our sample of research network participants hear more often and earlier about trends in the medical literature than their peers. With regards to the difference between language areas, it is possible that diffusion of ‘Smarter Medicine’ has been more effective in German-speaking areas, or that the ‘Choosing Wisely’ initiative in Germany, ‘Gemeinsam klug entscheiden’, has raised awareness in Switzerland [22].

We focused on physician attitudes towards the top five list and self-reported practices, one of three possible areas when assessing the impact of campaigns to decrease use of low-value care [23]. If GPs agree with the recommendations, they may be more likely to follow them in practice, as there was a correlation between giving agreement scores less than eight and GPs reporting that they follow fewer recommendations.

The association between having an in-practice pharmacy and following more recommendations may be primarily a marker for language area, as the overwhelming majority of GPs with an in-practice pharmacy are located in German-Switzerland. The only medication-related recommendation was for discontinuing PPIs, and there was not a statistically significant difference when examining responses of those with and without a pharmacy.

Concerning following individual recommendations, over two-thirds of GPs said that they rarely or never go against the recommendation for each clinical situation, except for lowering the dose or stopping proton pump inhibitors (PPIs). There is extensive evidence associating the chronic use of PPIs, especially at high doses, with various adverse outcomes such as increased infections, anaemia and fractures [24–26]. Despite multiple guidelines advocating for conservative, short-term use of PPIs in most situations, their chronic use appears to be increasing rapidly [25,26]. PPIs have few immediate side effects and can reduce dyspepsia, an extremely common and usually benign symptom in the general population. These symptoms often rebound after chronic PPIs are stopped. A simple recommendation statement is likely to be inadequate to decrease meaningfully the use of PPIs. Prior research has shown that multiple interventions are often necessary to influence practice [7].

When looking more broadly at reasons for not following the ‘Smarter Medicine’ recommendations, several common themes emerge. Physicians often appear to feel pressure to yield to the patient or other provider requests. Non-specific low back pain offers an example where patients are often anxious and ordering imaging can be a tangible action that reassures both physicians and patients and may strengthen the therapeutic bond. Various hypotheses have been put forward to explain why American physicians appear to be deviating from guidelines for acute low-back pain even more than previously, such as inappropriate incentives and a plethora of different contradictory information sources [27]. In our results, patient request and GP desire for information were most frequently cited, while inadequate time and avoiding a professional error were less important. These reasons are similar to a Canadian family physicians survey where patient request was the primary driver [23], but different than an American survey where malpractice and safety concerns were more important [18].

### Strengths and limitations

The generalizability of our results may be limited because of selection bias on two levels. First, our response rate of 60% and use of an electronic survey may have a disproportionate number of GPs who are better informed or more favourable of the ‘Smarter Medicine’ campaign. Second, our participants were GPs who had already agreed to be part of a practice-based research network. However, the SPAM network was designed to be representative and appears to be reflective of practicing GPs based on demographic characteristics [16]. In addition, longer-term data should be collected to know whether this campaign has a lasting impact.

Our results are based on physician report and not actual practice, which could lead to overly optimistic reporting from physicians due to social acceptability bias. Future research could observe actual consultations or claims data. Early reports of the impact of the ‘Choosing Wisely’ campaign on physician prescribing in other countries have been mixed [28,29]. However, there is evidence that Switzerland has the lowest rates of antibiotic prescription in Europe and low use of imaging tests, suggesting that compliance may be quite high at baseline. Finally, the cross-sectional, observational nature of our results prevent us from drawing conclusions about the cause of our findings, and it remains unclear if interventions to increase GP agreement with the recommendations would result in more physicians following them. It is also unclear whether the high rate of agreement with the recommendations or adherence was temporally associated with the ‘Smarter Medicine’ campaign.

### Implications

These findings could have important implications as the ‘Smarter Medicine’ campaign moves forward. Given that awareness among GPs is already high, work will be needed to sustain interest and there may be a ceiling effect limiting the impact of future diffusion efforts. The focus should be on ensuring physician agreement, particularly for the limitation of PPI prescription. Other interventions to promote top five lists could help GPs redirect requests for low-value care, extend the list beyond five items, and explore the clinical scenarios in which GPs feel they need to go against the recommendations.

## Conclusion

Awareness of the ‘Smarter Medicine’ campaign, a Swiss extension of the worldwide ‘Choosing Wisely’ movement, and agreement with its recommendations, appear to be high among GPs. However, the recommendation to decrease dosing or stop PPI’s is not being followed consistently.
